# Occupational Skin Dermatitis among Healthcare Workers Associated with the COVID-19 Pandemic: A Review of the Literature

**DOI:** 10.3390/ijms24032989

**Published:** 2023-02-03

**Authors:** Yu Sawada

**Affiliations:** Department of Dermatology, University of Occupational and Environmental Health, 1-1, Iseigoaka, Yahatanishi-Ku, Kitakyushu 807-8555, Fukuoka, Japan; long-ago@med.uoeh-u.ac.jp

**Keywords:** occupational skin diseases, COVID-19, irritant contact dermatitis, allergic contact dermatitis, acne vulgaris

## Abstract

The skin is the outermost layer of the human body and is continually exposed to numerous external stimuli, which can cause unwanted skin irritation. Occupational skin diseases are the most prevalent form of work-related illness and are found in a variety of sectors, particularly healthcare. During the recent COVID-19 pandemic, healthcare professionals experienced a variety of unexpected, unusual occupational skin diseases associated with COVID-19-engaged employment. Because the clinical characteristics of these types of skin inflammation are unique, this review focuses on the characteristics of a large category of occupational workers, namely COVID-19-engaged healthcare professionals. Furthermore, we examined the potential pathogeneses of occupational skin disorders associated with COVID-19-engaged labor, as well as different preventative methods.

## 1. Introduction

The skin is the organ of the human body that is exposed to the widest range of external environmental stimuli. External factors such as trauma, noxious substances, and haptens amplify the cutaneous inflammatory response and cause different inflammatory cytokines to remove the invading materials so as to safeguard the host human body [1,2]. However, these inflammatory reactions result in unwanted excess skin inflammation [3], and the quality of life of the affected individuals is frequently decreased by painful skin inflammation or inflammatory responses.

Several cutaneous responses to COVID-19 vaccinations have been reported [4]. The most common include urticarial, maculopapular, morbilliform, or papulovesicular eruptions and chilblains, livedo and vasculitis, swelling at the locations of cosmetic fillings, varicella-zoster or herpes simplex eruptions, pityriasis-rosea-like responses, and COVID arm [5]. Other studies have also indicated that the development of psoriasis may occur after receiving a vaccine, in addition to the onset of lichen planus or atopic dermatitis and the exacerbation of pre-existing hidradenitis suppurativa or pemphigus vulgaris [6,7].

Additionally, it has been observed that SARS-CoV-2 can cause a range of clinical skin abnormalities. The most prevalent cutaneous patterns linked with COVID-19 are chilblain-like lesions, maculopapular lesions, urticarial lesions, vesicular lesions, and livedoid lesions. Erythema-multiforme-like lesions, skin features consistent with multisystem inflammatory syndrome in children and, less frequently, multisystem inflammatory syndrome in adults (MIS-A), and pityriasis rosea resistant to standard therapy are other skin symptoms related to SARS-CoV-2 infection [8,9]. Furthermore, COVID-19 cutaneous symptoms include maculopapular, chilblain-like, urticarial, vesicular, livedoid, and petechial lesions. Rashes are frequent in multisystem inflammatory syndrome in children, a novel and deadly health illness whose symptoms overlap with Kawasaki disease and is likely connected to COVID-19 [10].

Occupational skin diseases are representative of cutaneous inflammation [11,12,13], and during the spread of COVID-19 around the world [14,15], healthcare workers experienced a variety of skin conditions due to their duties in providing treatment for COVID-19 patients [16,17,18,19,20,21,22]. To obtain a better understanding of the evolving features of occupational skin diseases among healthcare professionals and to appropriately treat such skin disorders, this review provides an updated overview of the knowledge gathered from previous investigations [23].

## 2. General Characteristics of Occupational Skin Disorders

Previous statistical studies indicated that skin diseases are recognized as the most prevalent occupational health issue. In the United States, the yearly incidence was 67 cases per 100,000 employees in 1997 [24], and the incidence of occupational skin diseases was 13% [24]. However, updated product techniques and various chemicals have been developed in recent years, and the actual frequency of occupational skin diseases is speculated to be higher than expected.

Indeed, recent clinical studies showed that a higher frequency of occupational skin diseases was observed in various countries. A clinical study including 422 participants in Ethiopia found that the most prevalent skin disease was occupational contact dermatitis (31.5%) [25]. The reported symptoms were redness (28.5%) and burning (17.3%) [25]. The hand is often the afflicted body region, accounting for 22% of cases. The most common risk factor of occupational skin diseases was frequent handwashing (OR: 1.80, 95% confidential interval (CI): 1.10–3.20), followed by personal allergy history (OR: 2.37, 95% CI: 1.32–4.61). The absence of health and safety education increased the risk of occupational skin diseases (OR: 2.12, 95% CI: 1.12–2.25) [25], indicating that education about occupational skin diseases is important for the future prevention of skin diseases. 

As in the case of the occupational types, a Thailand study showed that wet labor (35.1%) was the most common form of employment resulting in occupational skin diseases, followed by office workers (24.7%), industrial workers (16%), and healthcare workers (13.4%) [26].

According to these results, it is critical to acquire up-to-date knowledge so as to fully comprehend the current trends of occupational skin diseases and their clinical characteristics according to each job type.

## 3. Healthcare Workers and Occupational Skin Disease

Healthcare workers are exposed to various stimuli that exacerbate occupational skin diseases. Because of the high frequency of handwashing using detergents and sanitizers, in addition to the usage of gloves for long periods of the day, healthcare workers are at a higher risk of acquiring occupational contact dermatitis [27]. Therefore, it is speculated that healthcare workers are at a higher risk of developing occupational skin diseases.

A retrospective observational study including 1402 Danish healthcare personnel showed that 30% of individuals had occupational contact dermatitis and 53.4% of patients had hand dermatitis [28]. Another study including 508 healthcare workers in Canada showed that 30.5% of participants had hand dermatitis [29].

Occupational skin diseases are also sensitive to other agents. Findings of patch tests of 2248 nurses with occupational contact dermatitis showed a higher incidence of positive reactions to thiuram mix in 6.7% of cases, potassium dichromate in 5.6% of cases, and methylchloroisothiazolinone/methylisothiazolinone in 4.4% of cases [30]. Furthermore, another study showed that 13 healthcare workers with hand dermatitis had allergic contact dermatitis sensitive to glutaraldehyde and also showed concurrent sensitivity to additional substances in 76.9% of cases [31], suggesting that healthcare workers may often be sensitized to other causative agents that enhance occupational skin diseases.

Occupational dermatitis is likely to affect the quality of life of healthcare workers. An investigation of 37 occupational skin dermatitis cases showed a deterioration in the skin-related quality of life (OR = 19.3) [32], indicating that healthcare providers often work with uncomfortable occupational skin diseases.

## 4. Occupational Skin Diseases among COVID-19-Engaged Healthcare Workers

Healthcare workers have a high chance of developing occupational hand dermatitis, which has become more widespread since the COVID-19 epidemic. Because of the heightened number of preventive operations and the high patient load associated with the infection, the pandemic promoted the implementation of hand hygiene activities and longer usage of personal protective equipment by healthcare professionals [33], leading to a high frequency of occupational contact dermatitis during the pandemic. 

A cross-sectional questionnaire study of 376 healthcare workers revealed that 280 respondents (or 74.5%) reported having unpleasant skin responses [34]. Notably, this percentage was significantly higher than the rates of adverse skin responses during the COVID-19 outbreak [34]. Dryness or scale eruptions (68.6%) and papules or erythema eruptions (60.4%) were the most frequently reported types of eruption [34]. The three most frequently afflicted locations were the hands (84%), cheeks (75%), and the bridge of the nose (71%) [34]. In a multivariate analysis, more severe epidemics among hospital workers (OR: 2.41) and the wearing of full-body PPE for more than six hours per day (OR: 4.26) were linked to an elevated risk of unpleasant skin responses [34].

In a single-center, cross-sectional study of 270 healthcare workers in an Irish hospital, 223 (82.6%) of the participants reported having irritant contact dermatitis symptoms [35]. The most frequently affected body part was the hands (76.5%), and dry skin was the most common symptom (75.4%). Almost all the healthcare workers (99.3%) practiced more frequent handwashing; however, 45.35% of those workers did not use emollients [35]. A past history of skin diseases increased the risk of irritant contact dermatitis among COVID-19-engaged health workers, and 24.7% of those with irritant contact dermatitis reported having had dermatitis in the past [35]. Participants with irritant contact dermatitis used PPE for 3.15 h on average compared to those without irritant contact dermatitis, who used PPE for 1.97 h [35]. It is critical to improve our knowledge of how irritant contact dermatitis is linked to COVID-19 in order to enhance the disease’s prevention and treatment among frontline healthcare workers.

### 4.1. The Types of Skin Eruption among COVID-19-Engaged Healthcare Workers

Questionnaire surveys were conducted in various countries and highlighted issues affecting clinicians related to the highly increased incidence of occupational skin diseases due to the COVID-19 pandemic and the characteristics of these types of skin eruption due to COVID-19-engaged work.

The most prevalent symptoms presenting among 33 healthcare workers who performed COVID-19 duties were skin flaking, which was seen in 24.7% of cases, scaling in 15.5% of cases, and swelling in 13.4% of cases [36]. Cheilitis simplex, or generalized lip dryness, was the most common type of cheilitis (63.64%). Angular cheilitis was observed in 36.36% of cases, and 15.15% of workers developed perioral involvement [36]. Dermatitis of the lips was seen in 30.30% of cases, which was attributable to N95 mask contact [36]. The most prevalent consequences were secondary infections in 27.3% of cases and hyperpigmentation in 18.2% of cases [36]. Spicy meals and hot beverages were the most prevalent aggravating factors (78.8%), followed by picking/peeling habits (51.5%) and N95-mask-related contact dermatitis (30.3%) [36]. 

Another investigation showed that 35.7% of healthcare workers were diagnosed with irritant contact dermatitis, 28.5% were diagnosed with allergic contact dermatitis, and 21.4% of workers developed sweat dermatitis [37]. N95 masks with thermoelastic polymer straps were the most frequently utilized type of mask among 35.7% of healthcare workers [37]. Latex was the most common strap material, causing dermatoses in 28.5% of cases. Pre-existing dermatoses were identified in 50% of cases, including atopic dermatitis or seborrheic dermatitis [37].

A questionnaire survey of over 6,886 Swedish healthcare workers was used to assess the incidence of hand and facial complications [38]. In comparison to those who were not directly involved in COVID-19 care, workers caring for COVID-19 patients experienced more wet work and exposure to face masks, as well as a higher frequency of hand eczema, which was observed in 36% of cases, and facial skin disease, observed in 32% of cases, during a 1-year observation period [38]. The frequency of hand eczema and facial skin disease was noticeably higher among healthcare professionals who participated in COVID-19 care. In the past 12 months, the ORs for hand eczema and face skin diseases were 1.27 (95% CI: 1.06–1.53) and 1.34 (95% IC: 1.11–1.62), respectively. Acne (11%) and eczema (9%) were the most frequent cutaneous issues affecting the face [38].

A study including 292 Italian healthcare professionals reporting on dermatological diseases showed that 18.49% of workers had eczema, 13.01% had acne, and 16.44% had seborrheic dermatitis [39]. A previous history of inflammatory skin disorders, female sex, and continuous PPE usage significantly increased the risk of developing occupational skin diseases [39].

A study of thirty-two healthcare workers in the United Kingdom showed that 75% of workers had occupational skin diseases following the COVID-19 pandemic, and the most frequent occupational skin diseases were contact dermatitis, which appeared in 43.8% of cases, and folliculitis/acne in 18.8% of cases [40].

An online questionnaire was provided to healthcare workers using full PPE in a self-reported study, and the prevalence of occupational dermatitis was 61.9% based on 10,287 responses from a Danish population during the COVID-19 pandemic [41]. Dermatological problems were observed in a considerably larger proportion of healthcare workers with chronic skin disorders (71.6%) compared to those with no history of previous chronic skin diseases (59.7%) [41]. Healthcare workers wearing full PPE for more than 6 h each day had a greater risk of developing occupational skin diseases [41].

In a Korean population, the most frequent skin disease among 330 healthcare workers during the pandemic was the novel onset of contact dermatitis (33.9%), followed by new-onset acne (17.0%) and the exacerbation of pre-existing acne (17.0%) [42]. The use of masks on a daily basis was shown to be substantially linked with new-onset contact dermatitis. Wearing masks for a longer period (>6 h per day) and using cotton masks substantially exacerbated acne flare-ups [42].

A meta-analysis of the influence of mask wearing showed that facial skin diseases were observed in 55% of cases. Acne was the most prevalent skin disease, and 31% of workers had acne, followed by face dermatitis (24%), itch (30%), and pressure injuries (31%) [43].

Unexpected dermatosis, rosacea, and perioral dermatitis have been identified as skin eruptions associated with COVID-19-engaged work. A total of 1017 healthcare personnel participated in the survey, which showed that rosacea exacerbation rose following the COVID-19 pandemic, being seen in 39.1% of patients [44]. A retrospective study comparing the incidence of perioral dermatitis diagnosed by dermatologists before and during the COVID-19 pandemic based on 15,177 patients showed that mask use was associated with a 2.54-times-increased chance of developing perioral dermatitis (95% CI: 1.98–3.25), according to a multivariable logistic regression analysis [45].

These findings indicate that occupational skin diseases are on the rise, having received great attention from clinicians during the COVID-19 pandemic across the world, and the incidence of occupational skin disease appears to be similar in different countries. Notably, the incidence of occupational skin diseases was found to be increased compared to that among pre-COVID-19 pandemic healthcare workers.

### 4.2. The Factors Influencing Occupational Skin Diseases in the COVID-19 Pandemic

There are several factors that posed a risk of occupational skin diseases during the COVID-19 pandemic, such as PPE including face masks and eye goggles, frequent washing of hands, and hand sanitizers.

A comprehensive evaluation was carried out to identify the prevalent PPE-related dermatoses, afflicted body locations, and occupational contact materials. Sixteen studies were included, with a total of 3958 individuals. Xerosis, pressure-related erythema, and contact dermatitis were the most frequent dermatoses, primarily affecting the face and hands [46]. The most frequently implicated occupational contact materials were increased hand hygiene practice (48.4%) and the use of gloves (34.2%), N95 masks (26.9%), and goggles or face shields (21.1%) [46]. Consistently, the full cohort was examined, and the most frequently affected body areas were the face and hands [46]. The trunk and legs were unaffected.

#### 4.2.1. The Influences of Hand Sanitizers and Handwashing

The incidence of hand eczema was dramatically enhanced by handwashing at least eight to ten times each day (relative risk: 1.51) [47]. Wearing occlusive gloves, in addition to handwashing and alcohol hand sanitization, increases the chance of developing hand eczema [48]. The skin becomes macerated as a result of hyper-hydration caused by the gloves, which enhances the penetration of cleansers and alcohol sanitizers [49].

Following the application of ethanol to the skin, there was an increase in transepidermal water loss (TEWL), which was later linked to the extraction of lipids [50]. At varied ethanol concentrations, several studies noted variations in stratum corneum electrical resistance and conductivity, as well as variations in stratum corneum lipid melting temperatures [51].

#### 4.2.2. The Influences of Mask Wearing

Wearing a mask changes the biophysical properties of the skin, and N95 respirators were found to cause higher skin responses than medical masks [52]. To investigate the short-term effects of N95 masks and medical masks on skin physiological parameters, a randomized crossover study including 20 Chinese volunteers showed that wearing protective equipment enhanced the skin moisture, TEWL, and pH considerably. With the use of a mask, sebum excretion increased in the case of both covered and uncovered skin [53].

In Wuhan, healthcare personnel completed 61 valid surveys, showing that basal bridge scarring (68.9%) and facial itching (27.9%) were the most prevalent adverse skin responses among healthcare workers wearing N95 masks [54]. Dry skin (55.7%), itching (31.2%), and rash (23.0%) were the most prevalent adverse skin responses to wearing latex gloves [54]. Dry skin (36.1%) and itching (34.4%) were the most prevalent adverse skin responses among healthcare workers using protective equipment [54]. Most healthcare workers experienced unpleasant skin responses after wearing PPE for an extended period of time. The rate of adverse skin responses to the N95 mask was 95.1%, with response rates of 88.5% for latex gloves and 60.7% for protective garments [54], indicating that N95 mask wearing is one of the triggers of occupational contact dermatitis among COVID-19-engaged healthcare workers.

In addition, several materials contained in the mask are causative agents of skin dermatitis. An investigation of 21 pieces of facial PPE (11 N95 respirators and 10 surgical masks) showed that the most prevalent substance found in face PPE is polypropylene [55]. The nosepieces of the majority of masks contain aluminum [55]. Nickel was detected in two surgical masks [55]. Irritating contact dermatitis, allergic contact dermatitis, acne, and contact urticaria are all examples of facial PPE dermatoses. 

The mask quality also influences the development of skin inflammation. A study including 12 participants showed that patients with non-CE (European conformity mark)-approved masks had a higher, statistically significant prevalence of facial dermatoses (non-CE-approved mask: 45.1%, CE-approved mask: 8.7%) and irritating contact dermatitis (non-CE-approved mask: 16.9%, CE-approved mask: 0%) [56].

B. simplex, cereus, oleronius, and pumilus strains of D. folliculorum have been identified and linked to mask-related dermatosis [57,58,59,60,61]. A population-based study including 86 Japanese patients presented a method for acquiring key data on rosacea and comparable disorders, including climatic conditions and Demodex mites, in circumstances where face masks were required due to the coronavirus disease 2019 pandemic. Acaricidal and antibacterial drugs were used for the treatment. Rosacea and demodicosis improved as a result of the therapy [62].

#### 4.2.3. Decreased QOL among COVID-19-Engaged Healthcare Workers

A questionnaire study that enrolled healthcare workers using PPE showed that the DLQI was considerably lower among women and was associated with a higher rate of skin issues caused by PPE [36]. PPE usage enhanced the severity of previously identified skin illnesses and allergies in 22.3% of the respondents [36], and acne became more prevalent due to mask wearing [36].

## 5. The Prevention of Occupational Skin Diseases among COVID-19-Engaged Healthcare Workers

Based on the exacerbating factors of occupational skin diseases, several prevention methods have been postulated, such as the wearing of masks and the importance of skin moisturization.

Direct contact with the prevalent skin inflammation site can be reduced by using facial skin protectants based on the qualitative fit testing of N95 masks, which reduced skin dermatitis such as Cavilon film [63]. In addition, a simple method of supporting the surgical mask ties using a regular hospital wristband can help to prevent retroauricular dermatitis [64].

Hand moisturization is also helpful as a means to improve hand eczema in cases of occupational dermatosis. The majority of occupational hand dermatitis cases are irritant contact dermatitis, which is caused by continuous contact with hand hygiene products. A tertiary hospital conducted a trial in which four workplace interventions were put into place: (a) changing the currently used alcohol-based hand rub for a milder one, (b) switching the alcohol-based hand rub for gentle handwashing products, (c) temporarily changing the job to include less clinical work, and (d) switching from latex gloves to nitrile gloves [65]. A total of 21 participants enrolled in this study showed a significantly improved hand condition. [65]. Therefore, healthcare workers with irritant hand dermatitis may recover more quickly with the use of workplace treatments such as switching to milder versions of irritating hand hygiene products and temporarily reducing skin inflammation.

To clarify the importance of intervention in these cases, 230 nursing students were separated into two study groups, including an intervention group and control group, to assess the efficacy of a brief educational intervention program in preventing occupational hand eczema among nursing students [66]. Three months later, 59.52% of the control group participants and just 11.34% of the intervention group participants reported having experienced hand eczema in the preceding three months. The study found that hand eczema was a prevalent occupational dermatosis affecting medical professionals, even during apprenticeship. The prevalence of occupational contact dermatitis can be decreased by early prevention training programs.

## 6. Patch Testing and Causative Agents

A patch test is a diagnostic technique used to identify the precise chemicals that aggravate an allergic patient’s skin condition. Patch testing assists the assessor in determining which drugs may cause the individual’s delayed-type allergic reaction. We specified the materials that are included among allergically causative materials in order to better comprehend the representative causative agents of these variables.

### 6.1. Gloves and Causative Agents

According to patch test results of healthcare professionals who developed hand dermatitis after donning gloves, 86% of those tested responded to 1,3-diphenylguanadine, 84% to carba-mix, and 30% to thiuram mix [67], showing that the most prevalent relevant allergens were carba-mix and thiuram mix.

### 6.2. Hand Sanitizer and Causative Agents

Benzalkonium chloride, formerly assumed to be an irritant primarily found in nature, was recently linked to higher sensitization rates [68]. More common allergens include tocopherol, fragrance, propylene glycol, benzoates, and cetyl stearyl alcohol [69]. Preservatives that emit formaldehyde and cocamide diethanolamine (DEA) have also been identified as allergens in hand cleansers [70].

### 6.3. Masks and Causative Agents

Another causal factor is the rubber accelerators in mask elastic bands [71]. Recently, formaldehyde in a polypropylene surgical mask and polyurethane sponge in an N95 mask were identified as allergic contact dermatitis agents [72]. Nickel and cobalt in N95 masks have been discovered to be potential allergens [73].

## 7. Summary of, and Insights into, Dermatosis Associated with COVID-19-Engaged Work

We summarize the representative forms of dermatosis associated with COVID-19-engaged work in Table 1 and describe possible insights into their contributions to the development of these representative dermatosis forms. 

**Figure 1:**
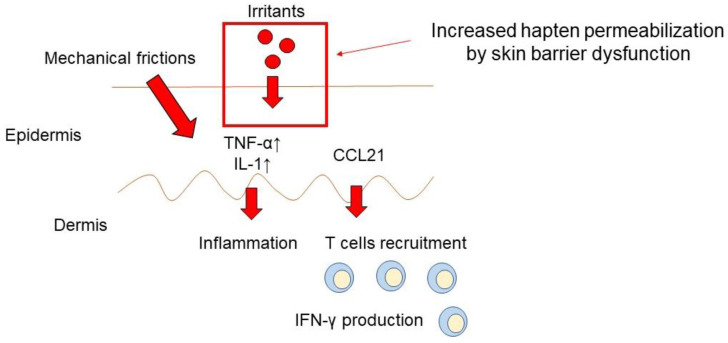
The pathogenesis of irritant contact dermatitis.

Irritant contact dermatitis is an inflammatory reaction that develops when keratinocytes are destroyed due to the toxicity of the contact source, resulting in the release of lysosomes and different cytokines [74,75,76] (Figure 1). By stimulating the skin above a specific threshold, irritant contact dermatitis enables the development of non-specific inflammation, even at the time of initial exposure. Irritant contact dermatitis caused by regular exposure to low-toxic compounds such as soap or chemicals has been on the rise in recent years, accounting for 70% of occupational skin diseases [77]. The pathogenesis of irritant contact dermatitis depends on endogenous and exogenous factors, such as individual skin and environmental or irritant characteristics [78]. Contact with irritants results in a non-specific response that compromises the function of the skin’s protective barrier, resulting in immediate cellular damage to the epidermis and triggering the production of proinflammatory mediators [78]. Irritant contact dermatitis is also triggered by immunological reactions. Keratinocytes play a crucial role as mediators that produce pro-inflammatory cytokines and increase the production of cell adhesion molecules and major histocompatibility complex II antigens in response to the breakdown of the skin barrier [79,80]. Additionally, irritants cause IFN-γ to amass in the skin, and the cancellation of the IFN-γ function by anti-TNF-α antibodies impairs the development of irritant contact dermatitis [81]. It has also been demonstrated that proinflammatory cytokines such as IL-1 and TNF-α, as well as the chemokine CCL21, which attracts naive T-lymphocytes to the skin, are increased in the skin during irritation responses [82]. The skin barrier function is also involved in the development of irritant contact dermatitis. Consistently, the filaggrin gene, which encodes a protein that is crucial for the function of the skin’s barrier, has recently been linked to an increased risk of developing chronic irritant contact dermatitis [83]. Therefore, the breakdown of the skin barrier through hand sanitization and mechanical irritation caused by mask wearing are the causative factors of irritant contact dermatitis. 

**Figure 2:**
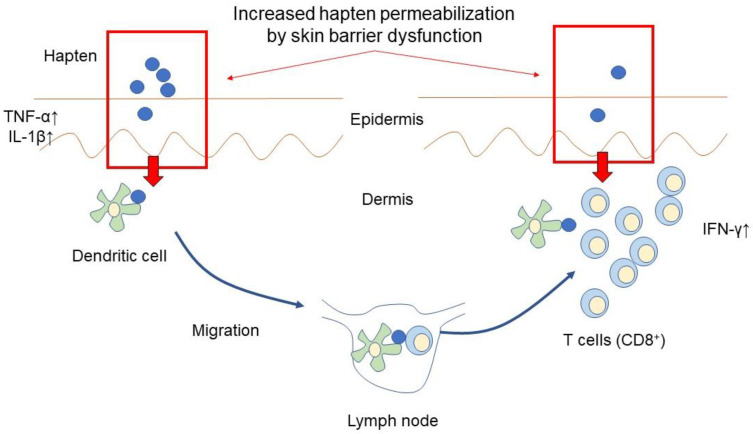
The pathogenesis of allergic contact dermatitis.

In allergic contact dermatitis, the pathophysiology is divided into two stages: sensitization and elicitation [84,85] (Figure 2). The generation of a variety of inflammatory cytokines and chemokines, including IL-1β and TNF [86,87], boosts the activation of cutaneous dendritic cells and controls their intake of antigens in order to prepare them for maturation and entry into the draining lymph node [88,89]. In the draining lymph node, naïve T cells activated by antigens begin to differentiate and proliferate into effector T cells, instigating the proper course of an immune response that is specific to the antigen [90]. Re-exposure to antigens activates antigen-specific T-cell infiltration during the elicitation phase [84]. These antigen-specific T lymphocytes release inflammatory cytokines, including IFN-γ, which then boosts the local inflammatory response [91]. Based on these mechanisms, hapten-containing materials such as gloves, hand sanitizer, and masks are candidate risk factors for occupational allergic contact dermatitis among COVID-19-engaged workers. 

**Figure 3:**
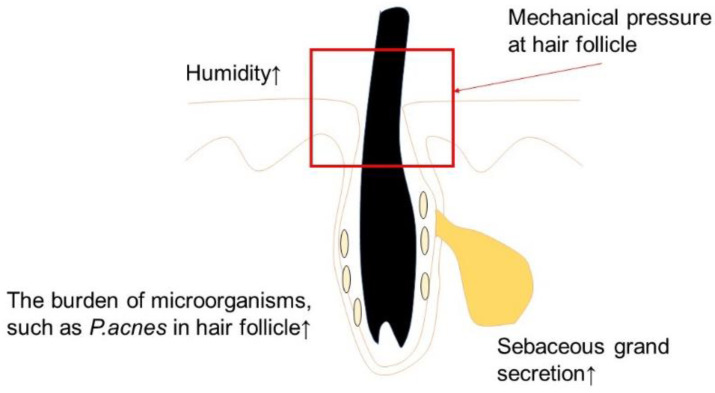
The pathogenesis of acne vulgaris.

Acne vulgaris has been observed and well recognized to be a response to the act of wearing a mask during the COVID-19 pandemic [92,93,94]. Face masks increase humidity and warm the area around the mask [95] (Figure 3). Increased humidity has the potential to aggravate acne vulgaris by occluding pores and harming the upper pilosebaceous unit of the skin [96]. Swollen keratinocytes can possibly result from sweating and higher humidity, leading to the blocking of follicles [96,97]. Inflammation also leads to the mechanical rupture of comedones caused by pressure and friction [96]. Additionally, alterations in the skin’s sebum composition and high humidity have significant impacts on bacterial growth. Microbiome dysbiosis is caused by heat, pH, and moisture due to mask wearing [98]. 

## 8. Conclusions

In relation to COVID-19-engaged work, we outlined the prevalence of occupational skin diseases among healthcare professionals. The aggravation variables concerning COVID-19-engaged work play crucial roles in preventing these forms of skin inflammation according to the pathomechanisms of occupational skin disorders, such as contact dermatitis. On the other hand, there are few strategies for controlling occupational skin diseases associated with coronaviruses among medical workers. Education and interventions for healthcare workers such as the use of moisturizer and specific dermatosis measures can be helpful in the prevention of skin eruption. In addition to basic treatments, environmental improvements are also required for the reduction in the number of occupational skin diseases. In addition, the estimation of the future risk of occupational dermatosis is also important for the management of occupational skin health. In particular, the representative causative agents in various forms of personal protective equipment and hand sanitizers have already been identified. Therefore, several routine surveillance methods, such as patch testing and previous allergic history examination, are necessary to avoid the risk of dermatosis associated with COVID-19-engaged work in the future. In the future, the development of skin-friendly masks, the management of hand moisturizer in the workplace, and education aiming to emphasize the importance of skin moisturization are essential for managing occupational skin diseases. 

The pathogenesis of irritant contact dermatitis depends on endogenous and exogenous factors, such as individual skin and environmental or irritant characteristics. Contact with irritants results in a non-specific response that compromises the function of the skin’s protective barrier, resulting in immediate cellular damage to the epidermis and triggering the production of proinflammatory mediators.

In addition to the allergens in personal protective equipment, highly frequent handwashing or hand sanitizer use enhances the skin barrier dysfunction, leading to the promotion of antigen infiltration into the skin and increasing the risk of sensitization or the elicitation of allergic contact dermatitis.

Face masks increase humidity and warm the area around the mask. Increased humidity has the potential to aggravate acne vulgaris by occluding pores and harming the upper pilosebaceous unit of the skin. Swollen keratinocytes can result from sweating and higher humidity, leading to blocked follicles. Inflammation also leads to the mechanical rupture of comedones caused by pressure and friction. Additionally, alterations in the skin’s sebum composition and high humidity have significant impacts on bacterial growth, in addition to skin surface alterations, such as changes in pH and temperature.

## Figures and Tables

**Table 1:** Summary of dermatosis associated with COVID-19-engaged work.

Eruptions	Causative Factors	Possible Mechanism
Contact Dermatitis	Gloves [48]	Allergic hapten
	Hand sanitizer [47].	Allergic hapten Skin barrier dysfunction
	Mask [54]	Mechanical irritation Allergic hapten
Acne Vulgaris	Mask [42]	Alteration in the local skin environment, e.g., the microbiome, temperature, moisture, mechanical follicular blocking
